# Prognostic Significance of Post-Operative Morbidity Severity Score After Potentially Curative D2 Gastrectomy for Carcinoma

**DOI:** 10.1007/s11605-018-3787-9

**Published:** 2018-05-15

**Authors:** Arfon Powell, Alexandra Harriet Coxon, Neil Patel, David Chan, Adam Christian, Wyn Lewis

**Affiliations:** 1 Division of Cancer and Genetics, Cardiff University, University Hospital of Wales, Heath Park, Cardiff, Wales UK; 2 Department of Surgery, Cardiff & Vale University Health Board, Cardiff, Wales UK; 3 Department of Pathology, Cardiff & Vale University Health Board, Cardiff, Wales UK

**Keywords:** Gastric cancer, Morbidity, Complications, Survival

## Abstract

**Background:**

Survival and relapse after gastric cancer surgery are largely attributed to tumor biology and surgical radicality; yet, other prognostic factors have been reported, including respiratory sepsis and anastomotic leakage, but not global morbidity severity score (MSS). The hypothesis tested was that MSS would be associated with both disease-free (DFS) and overall survival (OS).

**Methods:**

Consecutive 373 patients undergoing potentially curative surgery for gastric adenocarcinoma between 2004 and 2016 in a UK cancer network were studied. Complications were defined prospectively as any deviation from a pre-determined post-operative course within 30 days of surgery and classified according to the Clavien-Dindo severity classification (CDSC). Primary outcome measures were DFS and OS.

**Results:**

Post-operative complications were identified in 127 (34.0%) patients, which was associated with 9 (2.4%) post-operative deaths. Five-year DFS and OS were 35.9 and 38.5% for patients with a post-operative complication compared with 59.5 and 61.5% in controls (*p* < 0.001, *p* = 0.001, respectively). On multivariable DFS analysis, post-operative morbidity [hazard ratio (HR) 1.63, 95% confidence interval (CI) 1.06–2.50, *p* = 0.026] was independently associated with poor survival. On multivariable OS analysis, post-operative morbidity HR 2.25 (95% CI 1.04–4.85, *p* = 0.039) and CDSC HR 1.76 (95% CI 1.35–2.29, *p* < 0.001) were independently associated with poor survival. These associations were also observed in patients with TNM stage I and II disease with morbidity HR 7.06 (95% CI 1.89–26.38, *p* = 0.004) and CDSC HR 2.93 (95% CI 1.89–4.55, *p* < 0.001) offering independent prognostic value.

**Conclusion:**

Post-operative CDSC was an important independent prognostic factor after potentially curative gastrectomy for carcinoma associated with both DFS and OS. Prehabilitation strategies to minimize complications are warranted.

**Electronic supplementary material:**

The online version of this article (10.1007/s11605-018-3787-9) contains supplementary material, which is available to authorized users.

## Introduction

Positive versus negative outcomes after surgery are now commonly cited as the definitive measure of surgeon level competence, and certainly, any reasonable observer would surely agree that fatal complications, though uncommon, represent an important measure of outcome at the surgeon, hospital unit, and network level. In contrast, early post-operative morbidity has by tradition been considered to constitute a temporary blip in progress, with no long-term adverse sequelae, other than an associated prolonged duration of hospital stay, and associated short-term, poorer quality of life. Yet, some have contended that such morbidity, particularly after complex major gastrointestinal surgery, is associated with longer-term prognosis, disease relapse, and even cumulative survival.^1^^-^3

In global terms, gastric cancer is the third leading cause of cancer-related death, accounting for some 740,000 deaths annually.^4^ Surgery remains the only potentially curative treatment, but recurrence and metastasis occur in as many as 20 to 60% of the patients, and survival remains poor even after curative resection. Moreover, such surgery is complex major in nature, inherently high risk, with operative morbidity and mortality cited in the most recent UK National Oesophagogastric Cancer Audits^5^,^6^ to be 19.4 and 1.9%, respectively. Gastric cancer relapse and survival are largely attributed to tumor biology, aggressiveness, and the radicality of the surgery^7^, but other prognostic factors have also been reported, in particular anastomotic leakage^3^ and sepsis, after surgery for advanced gastric cancer. Why anastomotic leakage affects prognosis remains open to speculation; however, it has been argued that prolonged inflammatory response may promote the metastasis of residual tumor cells. It might also be argued that any stimulus provoking a systemic inflammatory response results in similar adverse outcomes. The aim of this study was to determine if overall post-operative morbidity severity classification might influence prognosis. The hypothesis was that the Clavien-Dindo morbidity severity classification would be associated with both disease-free and overall survival. The setting was a regional UK cancer network serving a population of 1.8 million.

## Method

### Patients

In order to test the hypotheses proposed in this study, a single cohort was developed and included patients with radiological TNM stage I to III, who following staging were deemed to have potentially resectable gastric cancer between January 2004 and December 2016. All patients were managed by a multidisciplinary team with an interest in gastric cancer and included surgeons, oncologists, radiologists, anesthetists, and pathologists. Preoperative staging involved computed tomography (CT) of the thorax, abdomen, and pelvis, including staging laparoscopy when considered appropriate, in order to facilitate individually patient-tailored management plans. Selective use of neoadjuvant chemotherapy was adopted following publication of the Medical Research Council Adjuvant Gastric Infusional Chemotherapy (MAGIC) Trial^8^ in the latter part of the study and was prescribed to 74 patients with minimal comorbidities who were deemed to have relatively advanced disease and would benefit from down-staging of the tumor prior to surgery. Chemotherapy was administered for 3 or 4 cycles preoperatively and post-operatively. Each cycle consisted of epirubicin (50 mg/m^2^) by intravenous bolus, cisplatin (60 mg/m^2^) as a 4-h infusion on day one, and 5-fluorouracil (200 mg/m^2^/day) daily by a continuous intravenous infusion.

The type of surgery for gastric cancer was determined by the anatomical location of the tumor; subtotal gastrectomy was performed in patients with antral tumors, and total gastrectomy was performed in patients with tumors of the cardia (Siewert type III), body, and linitis plastica. A modified extended D2 lymphadenectomy (preserving pancreas and spleen where possible) was performed and the operative approach was open in all cases. In 2010, an enhanced recovery after surgery program was introduced, the details of which have been described previously.^9^

Ethical approval was sought, but the chair of Cardiff & Value University Health Board ethics committee confirmed that individual patient consent was not required to report clinical outcomes alone, and no formal approval was necessary.

### Clinicopathological Characteristics

Tumors were staged using the seventh edition of the AJCC/UICC-TNM staging system.^10^ Pathological factors were recorded from pathology reports issued at the time of surgery and included tumor differentiation, vascular invasion, margin status, and the number of lymph nodes with and without metastasis.

Complications were defined prospectively as any deviation from a pre-determined post-operative course within 30 days following surgery. Patients undergoing a total gastrectomy underwent a gastrograffin swallow on post-operative day 5 to 7. Complications were diagnosed clinically based on observation, examination, and supplementary investigations including but not limited to blood testing (hematology and biochemistry), radiology, and microbiology. Once identified, complications were classified according to the Clavien-Dindo severity classification (CDSC).^11^ Grade I includes patients with any deviation from normal post-operative course. Grade II complications are treated solely by medicinal therapies. Grade III complications require physical intervention. Grade IV complications are deemed life threatening requiring admission to the critical care unit. Grade V represents post-operative death.

Patients were followed up at regular intervals of 3 months for the first year and 6 months thereafter. At each visit, patient underwent physical examination and blood analysis (hematology and biochemistry). Endoscopy and CT were performed when recurrent disease was suspected. In the event that patients developed symptoms suggestive of recurrent disease, investigations were undertaken sooner. Follow-up surveillance was conducted for 5 years or until death whichever was sooner. Death certification was obtained from the Office for National Statistics via Cancer Network Information System Cymru (CaNISC). Patterns of recurrence were defined as locoregional, distant (metastatic), or both locoregional and distant, when both were diagnosed at the same time. The time of recurrence was taken as the date of the confirmatory investigation.

### Statistical Analysis

#### Justification of Sample Size

Sample size calculations were based on a pre study literature survey of (CRUK cancers statistics reference), which indicated that the baseline 5-year survival rate of patients diagnosed with stage I gastric cancer was expected to be 80%, compared with 60% in patients with stage II gastric cancer, and a 15% difference in survival would be a realistic expectation. Thus, a minimum of 276 patients were to be studied, providing 80% power to detect such a difference with *p* < 0.05.

#### Methods of Data Analysis

Grouped data were expressed as median (range), and non-parametric methods were used throughout. Patient demographics were analyzed between the treatment modalities by means of *χ*2 or non-parametric tests including Mann-Whitney *U* test. These tests were also employed in the analysis of disease recurrence and time to recurrence for the treatment groups. Overall survival was calculated from time of diagnosis to the date of death. This approach was adopted in previous randomized trials^8^ to allow for the variable interval to surgery following diagnosis, depending on whether neoadjuvant therapy was prescribed. Disease-free survival was measured from the date of surgery until the date of recurrence or date of censoring. The median follow-up was 60 months (range 6 to 60), with 273 patients (73%) followed up for 5 years or until death. No patients were lost to follow-up. Cumulative survival was calculated according to the method of Kaplan and Meier; differences between groups were analyzed with the log rank test. Univariable analyses examining factors influencing survival were examined initially by the life table method of Kaplan and Meier, and those with associations found to be significant (*p* < 0.100) were retained in a Cox proportional hazards model using forward conditional methodology to assess the prognostic value of individual variables. All statistical analysis was performed in SPSS® (IBM® SPSS® Statistics v23.0.0.0, IBM Corporation, Armonk, New York, USA) with extension R.

## Results

### Patients, Clinicopathological Factors, and Post-Operative Morbidity

In total, 373 patients were identified who underwent surgery for gastric cancer. The complete baseline characteristics of all clinicopathological variables studied can be found in Table 1. The median age for patients undergoing resection was 69 years (inter-quartile range (IQR) 55–83) with the majority (43.7%) aged between 65 and 75 years. The majority of patients were male (68.1%), had distal cancers (43.7%), and were lymph node positive (55.2%). The median lymph node yield was 16 (range 3–64) with an inter-quartile range of 13. Neoadjuvant chemotherapy was prescribed to 74 patients (19.8%), and 78 patients (20.9%) received post-operative adjuvant chemotherapy (Table 1). There were 127 (34.0%) patients who developed post-operative complications, which were associated with 9 (2.4%) post-operative deaths within 90 days of surgery and 4 (1.1%) within 30 days of surgery. There were 74 (19.8%) infective and 22 (5.9%) non-infective complications (Table 2). Post-operative complications were associated with proximal tumor location (*p* = 0.013), higher pN category (*p* = 0.016), higher pTNM stage (*p* = 0.038), vascular invasion (*p* = 0.008), higher R1 status (*p* < 0.001), but not neoadjuvant (*p* = 0.634) or adjuvant (*p* = 0.804) therapy (Table 3). The median in-hospital length of stay (LOS) was 13 days (IQR 5–21). The median in-hospital LOS for CDSC 0 was 11 days (IQR 7–15), CDSC 1 was 16 days (IQR 8–24), CDSC 2 was 17 days (IQR 9–26), CDSC 3 was 24 days (IQR 10–38), CDSC 4 was 26 days (IQR 0–92), and CDSC 5 was 9 days (IQR 0–35). During follow-up, 93 patients (24.9%) developed cancer recurrence and 150 patients (40.2%) died. Two hundred sixty-seven patients (71.6%) were followed up for 5 years or until death (median 60 (6–60) months), and no patients were lost to follow-up.

**Table 1:** The relationship between tumor-related factors, overall survival, and disease-free survival in patients undergoing potentially curative resection for gastric cancer

Clinicopathological variables	Frequency n (%)	Disease-free survival	*p* value	Overall survival	*p* value
		Five-year survival rate (%)		5 year survival rate (%)	
Age (years)					
< 65 65–75 > 75 years	125 (33.5) 163 (43.7) 85 (22.8)	50.0 50.8 60.7	0.371	51.1 55.0 60.7	0.507
Sex					
Female Male	119 (31.9) 254 (68.1)	49.4 54.3	0.446	53.9 55.4	0.815
Tumor site					
Proximal Body Distal	124 (33.2) 86 (23.1) 163 (43.7)	35.4 65.7 57.3	< 0.001	39.0 65.7 59.7	0.002
T category					
1 2 3 4	88 (23.6) 28 (7.5) 134 (35.9) 123 (33.0)	83.8 81.3 48.8 30.2	< 0.001	83.8 87.5 50.5 33.3	< 0.001
N category					
0 1 2 3	167 (44.8) 76 (20.4) 72 (19.3) 58 (15.5)	73.1 49.0 31.3 15.0	< 0.001	74.6 51.0 33.3 20.0	< 0.001
Tumor stage					
I II III	100 (26.8) 119 (31.9) 154 (41.3)	85.1 58.1 25.5	< 0.001	85.1 62.4 27.4	< 0.001
Differentiation					
Well/moderate Poor	188 (50.4) 185 (49.6)	60.8 43.8	0.005	62.9 46.2	0.005
Vascular invasion					
No Yes	220 (59.0) 153 (41.0)	64.8 28.6	< 0.001	66.5 31.9	< 0.001
R status					
0 1	317 (85.0) 56 (15.0)	61.2 14.7	< 0.001	58.7 14.7	< 0.001
Neoadjuvant therapy					
No Yes	299 (80.2) 74 (19.8)	55.7 37.2	0.026	57.8 39.5	0.027
Adjuvant therapy					
No Yes	295 (79.1) 78 (20.9)	54.9 40.0	0.080	57.1 42.5	0.087
Post-operative morbidity			< 0.001		0.001
No Yes	246 (66.0) 127 (34.0)	59.5 35.9		61.5 38.5	
Infective complication			0.167		0.287
No Yes	299 (80.2) 74 (19.8)	54.6 43.5		56.4 47.8	
Non-infective complication			0.084		0.060
No Yes	351 (94.1) 22 (5.9)	53.8 27.3		56.1 27.3	
Clavien-Dindo classification			0.007		0.006
0 1 2 3 4 5	246 (66.0) 21 (5.6) 57 (15.3) 27 (7.2) 13 (3.5) 9 (2.4)	59.5 42.9 38.5 37.5 33.3 0.00		61.5 42.9 43.6 37.5 33.3 0.0

**Table 2:** The incidence of complications in patients undergoing potentially curative resection for gastric cancer

Classification of complications	
Infective complications	74 (19.8%)
Surgical site infection	
Anastomotic leak Duodenal leak Intra-abdominal abscess Wound Enterocutaneous fistula	18 (4.8%) 5 (1.3%) 3 (0.8%) 18 (4.8%) 2 (0.5%)
Extra-abdominal infection	
Pneumonia Urinary tract infection	24 (6.9%) 3 (0.9%)
Non-infective complications	22 (5.9%)
Cardiovascular	
Acute coronary syndrome Atrial fibrillation	2 (0.5%) 2 (0.5%)
Respiratory	
Pulmonary embolus Pulmonary edema Pleural effusion	2 (0.5%) 0 (0.0%) 4 (1.1%)
Miscellaneous	12 (3.6%)

**Table 3:** The relationship between post-operative morbidity and clinicopathological factors in patients undergoing potentially curative resection for gastric cancer

Clinicopathological variables	No complication *n* (%)	Complication*n* (%)	*p* value
Age (years)			
< 65 65–75 > 75 years	87 (35.4) 104 (42.3) 55 (22.4)	37 (29.4) 59 (46.8) 30 (23.8)	0.504
Sex			
Female Male	76 (30.9) 170 (69.1)	43 (34.1) 83 (65.9)	0.527
Tumor site			
Proximal Body Distal	71 (28.9) 66 (26.8) 109 (44.3)	53 (41.7) 20 (15.7) 54 (42.5)	0.013
T category			
1 2 3 4	68 (27.6) 16 (6.5) 84 (34.1) 78 (31.7)	21 (16.5) 12 (9.4) 49 (38.6) 45 (35.4)	0.106
N category			
0 1 2 3	125 (50.8) 46 (18.7) 40 (16.3) 35 (14.2)	43 (33.9) 29 (22.8) 32 (25.2) 23 (18.1)	0.016
Tumor stage			
I II III	76 (30.9) 78 (31.7) 92 (37.4)	25 (19.7) 40 (31.5) 62 (48.8)	0.038
Differentiation			
Well/moderate Poor	124 (50.4) 122 (49.6)	64 (50.4) 63 (49.6)	0.998
Vascular invasion			
No Yes	157 (63.8) 89 (36.3)	63 (49.6) 64 (50.4)	0.008
R status			
0 1	221 (89.8) 25 (10.2)	96 (75.6) 31 (24.4)	<0.001
Neoadjuvant therapy			
No Yes	196 (79.7) 50 (20.3)	103 (81.7) 23 (18.3)	0.634
Adjuvant therapy			
No Yes	196 (79.7) 50 (20.3)	99 (78.6) 27 (21.4)	0.804

### Relationships Between Post-Operative Complications and Disease-Free Survival

**Fig. 1:**
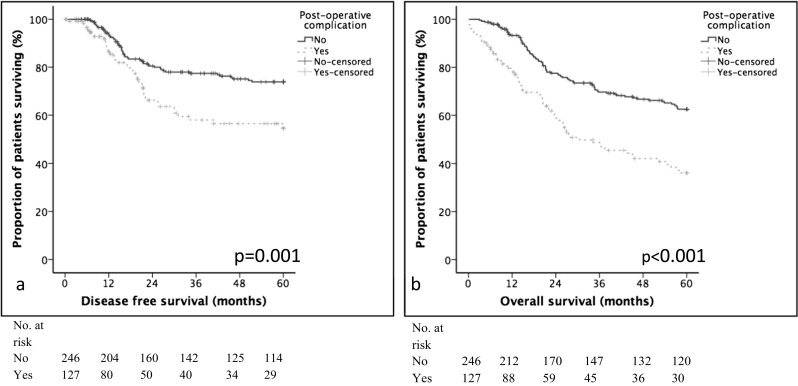
The relationship between post-operative complication, disease-free **a**, and overall survival **b**

A univariable analysis of factors associated with disease-free survival can be found in Table 4. On multivariable analysis, pT category (hazard ratio (HR) 1.80 (95% confidence interval (CI) 1.35–2.39, *p* < 0.001), vascular invasion (HR 1.87 (1.17–2.97), *p* = 0.008), R1 status (HR 2.14 (1.32–2.50), *p* = 0.002), and post-operative complication (HR 1.63 (1.06–2.50), *p* = 0.026) were independently associated with disease-free survival (Table 4). Five-year disease-free survival was 66.0% for patients without a complication compared with 34.0% for patients developing a post-operative complication (Table 1, Fig 1).

**Table 4:** Univariable and multivariable analysis of clinicopathological factors and complication markers: disease-free and overall survival

	Univariable	*p* value	Multivariable	*p* value	Univariable	*p* value	Multivariable	*p* value
	Disease-free survival				Overall survival			
	Hazard ratio (95% CI)		Hazard ratio (95% CI)		Hazard ratio (95% CI)		Hazard ratio (95% CI)	
Age (years) < 65/66–75/> 75)	0.77 (0.57–1.02)	0.072			0.89 (0.71–1.11)	0.298		
Gender (Female/male)	0.63 (0.41–0.96)	0.031		0.063	0.98 (0.69–1.39)	0.894		
Tumor site (Proximal/body/distal)	0.76 (0.59–0.97)	0.028		0.738	0.77 (0.63–0.94)	0.009	0.79 (0.65–0.96)	0.020
Neoadjuvant therapy (No/yes)	1.60 (0.99–2.58)	0.053			1.40 (0.95–2.07)	0.093		
Pathological factors								
T category (1/2/3/4)	2.19 (1.70–2.82)	< 0.001	1.80 (1.35–2.39)	< 0.001	1.93 (1.60–2.32)	< 0.001	1.67 (1.34–2.06)	< 0.001
N category (0/1/2/3)	1.82 (1.52–2.18)	< 0.001		0.081	1.66 (1.44–1.92)	< 0.001		0.068
TNM stage (I/II/III)	3.14 (2.24–4.41)	< 0.001		0.574	2.49 (1.95–3.18)	< 0.001		0.567
Differentiation (Moderate/poor)	2.14 (1.39–3.30)	0.001		0.369	1.86 (1.33–2.61)	< 0.001		0.089
Vascular invasion (No/yes)	3.36 (2.19–5.16)	< 0.001	1.87 (1.17–2.97)	0.008	2.96 (2.12–4.15)	< 0.001	1.76 (1.21–2.55)	0.003
R status (0/1)	4.19 (2.67–6.58)	< 0.001	2.14 (1.32–3.45)	0.002	3.27 (2.25–4.76)	< 0.001		0.619
Post-operative factors								
Adjuvant therapy (No/yes)	1.56 (0.96–2.53)	0.070			1.39 (0.93–2.06)	0.105		
Post-operative morbidity (No/yes)	2.17 (1.42–3.31)	< 0.001	1.63 (1.06–2.50)	0.026	2.28 (1.63–3.17)	< 0.001	2.25 (1.04–4.85)	0.039
Infective complication (No/yes)	1.30 (0.77–2.18)	0.328			1.42 (0.95–2.12)	0.084		
Non-infective complication (No/yes)	2.06 (0.90–4.72)	0.089			3.54 (2.03–6.17)	< 0.001		0.061
Clavien-Dindo classification (0/1/2/3/4/5)	1.27 (1.08–1.50)	0.004		0.197	1.50 (1.33–1.79)	< 0.001	1.76 (1.35–2.29)	< 0.001

**Fig. 2:**
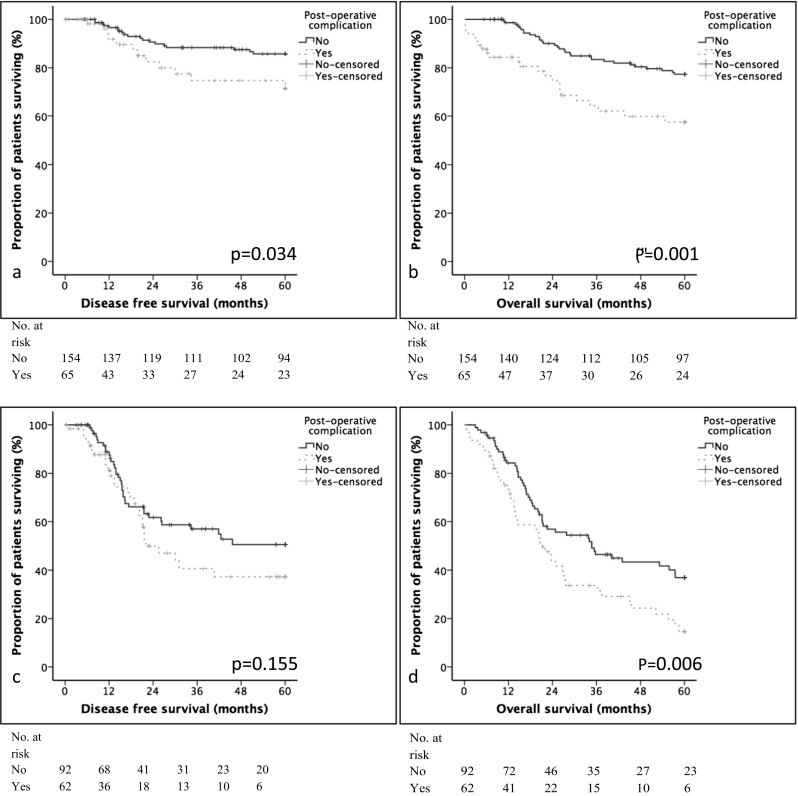
The relationship between post-operative complication, disease-free, and overall survival in patients with stage I and II gastric cancer. **a** Disease-free survival in patients with TNM stage I and II gastric cancer. **b** Overall survival in patients with TNM stage I and II gastric cancer. **c** Disease-free survival in patients with TNM stage III gastric cancer. **d** Overall survival in patients with TNM stage III gastric cancer

Patients were stratified into pTNM stage I and II, or pTNM stage III disease. Post-operative complication was associated with poorer disease-free survival (*p* = 0.038, Fig. 2a) in TNM stage I and II disease, but not in stage III disease (*p* = 0.158, Fig. 2c). On multivariable analysis, post-operative complication was not independently associated with poorer disease-free survival when confounded for other statistically significant factors (Tables 5 and 6).

**Table 5:** Clinicopathological factors and survival in stage I and II gastric cancer

	Univariable	*p* value	Multivariable	*p* value	Univariable	*p* value	Multivariable	*p* value
	Disease-free survival				Overall survival			
	Hazard ratio (95% CI)		Hazard ratio (95% CI)		Hazard ratio (95% CI)		Hazard ratio (95% CI)	
Age (years) < 65/66–75/> 75)	0.72 (0.44–1.17)	0.186			1.09 (0.76–1.56)	0.648		
Gender (Female/male)	0.99 (0.47–2.10)	0.981			1.21 (0.66–2.19)	0.540		
Tumor site (Proximal/body/distal)	0.61 (0.40–0.93)	0.022		0.231	0.67 (0.49–0.92)	0.013	0.68 (0.50–0.93)	0.014
Neoadjuvant therapy (No/yes)	2.29 (1.05–5.00)	0.037		0.662	1.92 (1.04–3.54)	0.037		0.961
Pathological factors								
T category (1/2/3/4)	1.89 (1.34–2.65)	< 0.001		0.845	1.48 (1.16–1.89)	0.001		0.902
N category (0/1/2/3)	2.92 (1.74–4.92)	< 0.001		0.310	1.64 (1.02–2.62)	0.039		0.711
TNM stage (I/II)	14.28 (3.41–59.85)	< 0.001	10.13 (2.37–43.31)	0.002	3.28 (1.73–6.23)	< 0.001	2.94 (1.51–5.74)	0.002
Differentiation (Moderate/poor)	1.47 (0.72–2.96)	0.288			1.07 (0.62–1.84)	0.814		
Vascular invasion (No/yes)	4.23 (2.08–8.58)	< 0.001	2.66 (1.26–5.59)	0.010	3.23 (1.88–5.56)	< 0.001	2.33 (1.29–4.20)	0.005
R status (0/1)	10.56 (4.65–23.96)	< 0.001	4.18 (1.76–9.93)	0.001	4.42 (2.06–9.46)	< 0.001		0.536
Post-operative factors								
Adjuvant therapy (No/yes)	1.80 (0.78–4.20)	0.172			1.75 (0.92–3.24)	0.090		
Post-operative morbidity (No/yes)	2.15 (1.04–4.43)	0.038		0.474	2.43 (1.42–4.18)	0.001	7.06 (1.89–26.38)	0.004
Infective complication (No/yes)	1.57 (0.67–3.63)	0.297			1.84 (1.00–3.38)	0.051		
Non-infective complication (No/yes)	1.43 (0.19–10.51)	0.727			3.20 (1.15–8.91)	0.026		0.864
Clavien-Dindo classification (0/1/2/3/4/5)	1.26 (0.93–1.70)	0.142			1.66 (1.37–2.00)	< 0.001	2.93 (1.89–4.55)	< 0.001

**Table 6:** Clinicopathological factors and survival in stage III gastric cancer

	Univariable	*p* value	Multivariable	*p* value	Univariable	*p* value	Multivariable	*p* value
	Disease-free survival				Overall survival			
	Hazard ratio (95% CI)		Hazard ratio (95% CI)		Hazard ratio (95% CI)		Hazard ratio (95% CI)	
Age (years) < 65/66–75/> 75)	0.85 (0.60–1.21)	0.374			0.86 (0.65–1.15)	0.302		
Gender (Female/male)	0.44 (0.27–0.72)	0.001	0.44 (0.26–0.72)	0.001	0.82 (0.54–1.24)	0.350		
Tumor site (Proximal/body/distal)	0.92 (0.69–1.23)	0.583			0.97 (0.77–1.23)	0.801		
Neoadjuvant therapy (No/yes)	1.13 (0.63–2.02)	0.683			0.94 (0.58–1.54)	0.814		
Pathological factors								
T category (1/2/3/4)	1.58 (0.92–2.71)	0.097			1.63 (1.06–2.53)	0.028		
N category (0/1/2/3)	1.26 (0.88–1.80)	0.202			1.30 (0.97–1.73)	0.079		0.051
Differentiation (Moderate/poor)	1.82 (1.06–3.13)	0.031	1.91 (1.11–3.30)	0.020	1.87 (1.20–2.91)	0.006	1.80 (1.15–2.84)	0.011
Vascular invasion (No/yes)	1.79 (1.05–3.07)	0.033	1.84 (1.07–3.15)	0.027	1.55 (1.02–2.37)	0.041		0.063
R status (0/1)	1.64 (0.96–2.78)	0.069			1.61 (1.05–2.47)	0.031		0.328
Post-operative factors								
Adjuvant therapy (No/yes)	1.13 (0.64–2.00)	0.676			0.95 (0.59–1.54)	0.844		
Post-operative morbidity (No/yes)	1.44 (0.87–2.38)	0.158			1.74 (1.16–2.60)	0.007		0.770
Infective complication (No/yes)	0.99 (0.52–1.91)	0.982			1.14 (0.69–1.89)	0.606		
Non-infective complication (No/yes)	1.44 (0.58–3.61)	0.432			2.74 (1.41–5.29)	0.003	2.24 (1.10–4.53)	0.025
Clavien-Dindo classification (0/1/2/3/4/5)	1.10 (0.90–1.35)	0.338			1.28 (1.10–1.49)	0.001	1.21 (1.04–1.42)	0.015

### Relationship Between Post-Operative Complications and Overall Survival

A univariable analysis of factors associated with overall survival can be found in Table 4. On multivariable analysis only pT category (HR 1.67 (1.34–2.06), *p* < 0.001), vascular invasion (HR 1.76 (1.21–2.55), *p* = 0.003), post-operative complication (HR 2.25 (1.04–4.85), *p* = 0.039), and Clavien-Dindo classification (HR 1.76 (1.35–2.29), *p* < 0.001) were independently associated with poor overall survival (Table 4).

Patients were stratified into TNM stage I and II, or TNM stage III disease. Post-operative complication was associated with poorer overall survival in TNM stage I and II disease (*p* = 0.001, Fig. 2b) and stage III disease (*p* = 0.007, Fig. 2d). On multivariable analysis, post-operative complication was only independently associated with poorer overall survival in patients with stage I and II disease (HR 7.06 (1.89–26.38), *p* = 0.004) (Table 5). Non-infective complications were associated with poorer overall survival in stage III disease (HR 2.24 (1.10–4.53), *p* = 0.025) (Table 5). Clavien-Dindo classification was also independently associated with poor overall survival in stage I and II disease (HR 2.93 (1.89–4.55); *p* < 0.001) and stage III disease (HR 1.21 (1.04–1.42); *p* = 0.015) (Table 6).

## Discussion

Surgical outcomes, in broad terms, have never been better or more transparent, despite the contemporary challenges of an increasingly elderly, comorbid, and sometimes frail population. High-risk surgical patients are at greater danger of post-operative complications, prolonged durations of hospital stay, and recovery in general blighted by a compromised quality of life. Moreover, patients diagnosed with cancer face and pose specific problems, including debility, weight loss, malnutrition, and anemia that may all impact outcomes. This is the first study to highlight the prognostic significance of post-operative morbidity severity classification after D2 gastrectomy for carcinoma. The principal findings supported the working hypothesis and showed that the one third of the patients who suffered any complication were 40% less likely to enjoy disease-free 5-year survival. The poorer survival associated with post-operative complications was independent of tumor histopathological stage, suggesting that treatment strategies aimed at minimizing complications may not only improve oncological outcome but also reduce lengths of hospital stay, improve quality of life, with allied consequent economic benefits for hospital services and prudent NHS health care.

Patients carrying significant comorbidities, poor functional performance, and higher risk assessment profiles are well recognized to suffer poorer post-operative quality of life and cumulative survival following abdominal surgery.^12^ Yet, only recently have the associations between pre-operative physiological functional status and post-operative disease recurrence been appreciated.^2^ Richards et al. reported in a cohort of patients from Glasgow, Scotland, with pTNM stage I–III colorectal cancer that poor pre-operative POSSUM scores were 50%more likely to develop post-operative complications and disease recurrence.^2^ Similarly, Kang et al. (South Korea),^13^ Zhang et al. (China), and 14 Inokuchi (Tokyo)^15^ have reported an association between pre-operative serum albumin and surgical complications in patients undergoing gastrectomy for cancer, which suggest that the systemic inflammatory response plays a pivotal role. Unfortunately, these latter reports focused only on serum albumin analyses and, other SIR biomarkers such as the C-reactive protein-based modified Glasgow Prognostic Score, may provide better prognostic information. Indeed, in a recent report comparing a raft of all serum-based inflammatory biomarkers, the modified Glasgow Prognostic Score (mGPS) was the only inflammatory marker independently associated with disease recurrence and overall survival.^16^ Measures that optimize the patients’ risk assessment profiles may offer the greatest therapeutic benefit, and the magnitude of these benefits has been signaled in a recent report form Barberan-Garcia et al. (Barcelona) who observed a 51% reduction in post-operative morbidity in patients undergoing intensive prehabilitation programs prior to major abdominal surgery.^17^

The adverse influence of global post-operative morbidity on overall survival has been reported previously. Li et al. from China^1^ reported significantly poorer 5-year overall survival in a cohort of 432 patients when morbidity occurred (21.8%) compared with controls (39.9%), which was independent of confounding factors (HR 2.5. *p* < 0.001). However, the value of overall survival as an outcome measure is relatively limited because of the inclusion of non-cancer-related deaths in the analysis, diluting the prognostic influence of cancer biology. In contrast, septic complications after surgery have been implicated in influencing disease-free survival.^18^,^19^ Both Tokunga et al.^18^ (*n* = 756) and Hayashi et al.^19^ (Japan) (*n* = 502) contended that sepsis was associated with poorer disease-free 5-year survival of the order of 20% (HR 2.22, *p* = 0.002) and 25% (HR 1.96, *p* = 0.013), respectively. The findings of this study are not in keeping with the above, but are in line with those reported by Nelen et from the Netherlands^20^ with sepsis associated with 33.1% poorer disease-free 5-year survival. Moreover, respiratory sepsis was fourfold greater in western cohorts (20%)^20^ when compared with eastern cohorts (5%).^19^

There are a number of inherent limitations and potential criticisms of this study. Data related to the patients’ race, body mass indices, and detailed comorbidity was not collected prospectively and was therefore not available for analysis as confounding factors. The patient cohort studied is a selected group (most had undergone a potentially curative R0 gastrectomy) and was not representative of all gastric cancer patients; indeed, only approximately one third of the South Wales patients undergo potentially curative resection.^21^ In contrast, the study has several strengths, benefiting from robust follow-up data with accurate causes and dates of death obtained from the office of national statistics; over 75% were followed up for at least 5 years or death. Patients were recruited consecutively from a single UK geographical region, and all had been treated by the same multidisciplinary team and group of specialist surgeons, using a standardized staging algorithm and operative technique, with extensive audited and published quality control. Moreover, the findings cannot be criticized because of poor surgical outcomes, which compare favorably with national trial and audit data in terms of post-operative morbidity and cumulative survival.^5^,^6^

In conclusion, the concept of surgical prehabilitation refers to an emerging field of research concerned with strategies to optimize the patients’ preoperative physical and psychosocial risk profiles, such that post-operative recovery trajectories are boosted, resulting in fewer complications, shorter durations of hospital stay, improved quality of life, and cost-effective prudent health care. Reports to date have focused on a heterogenous group of health interventions, applied within the care continuum, and occurring between the diagnosis and the start of surgical treatment. These have included, education, exercise, nutrition, and psychosocial approaches, focused not only the patient but also the patient’s family, with the aim of promoting health-related behavioral change that reaches beyond the immediate preoperative period into the future and longer term. Prehabilitation is the logical precursor to enhanced recovery programmes but should comprise more than just exercise. Nutritional and psychosocial well-being are also critical aspects of perioperative care and key components of prehabilitation programs. The preoperative period presents an opportunity to utilize a so-called “teachable moment” and emphasize the importance of positive lifestyle change such as smoking cessation. Future research efforts should explore combining and fusing prehabilitation with enhanced recovery programmes to catalyze additional improvements in outcomes. Moreover, cost-effectiveness evaluation should form part of future research. Prehabilitation in specialties with high-risk profiles will probably be associated with additional costs, though it is possible, if not likely, that such costs would be offset by improved outcomes such as shorter durations of hospital stay, fewer complications, and better quality of life. Finally and by tradition, prehabilitation programs are prescriptive and generic: employing a one size fits all philosophy. Bespoke personalized programs, related to the individual patients’ physiological, functional, psychosocial profiles, and including combinations of supervised and independent self-assessed exercises, delivered in the community rather than secondary care are likely to be associated with greater compliance and effect.

## Electronic Supplementary Material

ESM 1 (DOCX 246kb)
