# Supplementary material for: Prognostic Significance of Post-Operative Morbidity Severity Score After Potentially Curative D2 Gastrectomy for Carcinoma
Source: J Gastrointest Surg. 2018 May 15;22(9):1516–27. doi: 10.1007/s11605-018-3787-9 (PMC6132392; doi:10.1007/s11605-018-3787-9)

Supplementary figure 1. The association between Clavien-Dindo classification and disease-free survival


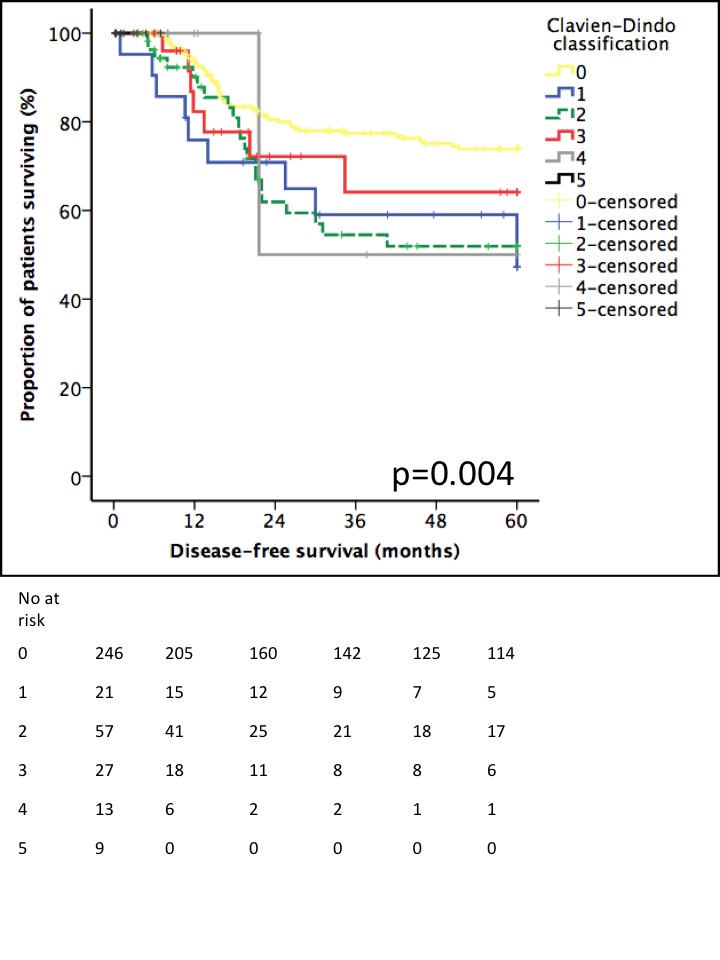


Supplementary figure 2. The association between Clavien-Dindo classification and overall survival


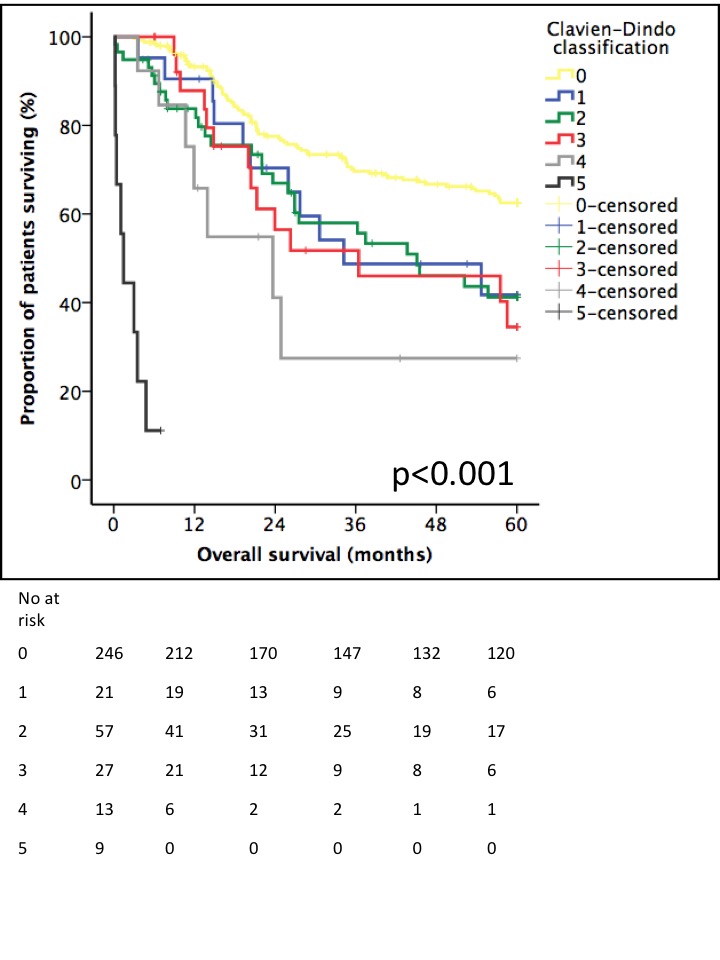

Supplement: Supplementary file 1 — (DOCX 246kb) [file 11605_2018_3787_MOESM1_ESM.docx]
